# Remedies and Their Uses

**Published:** 1906-12-01

**Authors:** 


### REMEDIES AND THEIR USES.

### Guaiacol and its Derivatives.

THE substances formed by introducing hydroxyl (OH) into the benzene ring are distinguished by their powerful antiseptic properties, and frequent attempts have consequently been made to find one suited to internal administration. Phenol, or carbolic acid, which is benzene in which one hydrogen atom is replaced by hydroxyl, is too toxic for internal use except in very small quantities. There are three dioxy-benzenes, differing in the relative positions of the substituted hydrogen atoms, and known as resorcin, pyrocatechin, and hydroquinone. Of these substances only resorcin has ever been given internally, and that is seldom now employed owing to its depressant action on the heart. A substance, however, which retains the antiseptic properties of its parent body, is guaiacol, a methyl derivative of pyrocatechin, the formula of which is

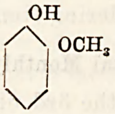

The use of this substance, however, is much restricted by its unpleasant taste and its irritant action on the mucous membrane of the stomach. It has, however, been found of some value, especially in the treatment of phthisis, bronchiectasis, and certain inflammatory conditions of the intestinal tract. Creosote, which is a mixture of guaiacol with certain higher cresols, can only be given in pills or capsules; there is a pharmacopœial mixture, but it is too nasty for the average patient. Recently several substances have been introduced which are intended to take the place of guaiacol; they are compounds which liberate that substance in the body, thus producing its therapeutic effects without its practical disadvantages. One of these is

### Guaiacophosphal, a neutral phosphite of guaiacol containing 92 per cent. of that substance and 7 per cent. of organic phosphorus. This is a white, straw-like crystalline body, soluble in water; it has a hot, but not burning, taste, and a slight odour. It may be given in capsules coated with gluten in doses of 2¼ grains several times daily, or in a solution, of which one to three teaspoonfuls may be administered in milk before meals every day. It is prepared by Clin and Co., 20 rue des Fossés Saint-Jacques, Paris. Another preparation is

### Thiocol, the guaiacol sulphonate of potassium, a white odourless powder soluble in water. It is bitter at first, but subsequently leaves a sweet taste in the mouth. It has been employed in various pulmonary conditions, including phthisis, whooping-cough, acute and chronic bronchitis, etc. It may be given in 5-grain doses thrice daily, gradually rising to 45 grains per diem. It does not, like guaiacol, interfere with digestion, but the limit of 45 grains a day should not be exceeded.

### Cresol.

This substance, chemically, is a glyceric ether of guaiacol, and has a pronounced antiseptic action. It differs from the ordinary guaiacol compounds, in that its antiseptic action does not appear to be due solely to the liberation of guaiacol. It is slowly decomposed in the intestine, and absorbed. It does not reappear in the urine conjugated as guaiacol sulphovinic acid, except to a very small extent, but the decomposition products have not as yet been fully worked out. It has a bitter taste, and is said to improve the appetite. It does not disturb digestion, and may be given in 3-drachm doses thrice daily to adults or half-drachm doses to children.

### Duotal.

This is guaiacol carbonate, and is supposed to be split up in the intestine, the guaiacol then exerting its antiseptic action, and being eliminated in combination mainly with sulphovinic acid in the urine. The dose is 5 to 15 grains. Like other guaiacol compounds, it has been used in phthisis and as an intestinal antiseptic. It contains more than 90 per cent. of guaiacol, of which half may be recovered in the urine, showing that free guaiacol is liberated to a considerable extent in the organism. It is prepared by Bayen of Elberfeld.

### Styracol.

This preparation is a cinnamic acid ether of guaiacol. Cinnamic acid has the formula:

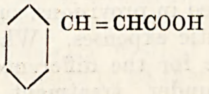

and has been employed under the name of hetol (sodium cinnamate) in the treatment of phthisis. Its action is to produce a local leucocytosis. In combination with guaiacol it forms an odourless crystalline body almost entirely insoluble in water, and possessing a not unpleasant taste. It may easily be dissolved in alcohol or chloroform. It is not markedly toxic. In the intestine the guaiacol is split off and excreted in the urine, combined with sulphovinic and glycuronic acids, while the cinnamic acid, reduced to benzoic acid, appears finally as hippuric acid. It appears to be more completely split up than any guaiacol compound; 84 per cent. of the guaiacol reappears in the urine, and thus it may be considered an effective substitute for ordinary guaiacol. It is prepared in tablets, which are meant to be bitten up, not swallowed whole, and in powder. Its poor solubility is against its use in a mixture, but the powdered drug may be made more palatable by the addition of an equal quantity of white sugar. The usual dose for adults is 15 grains three or four times a day; much larger doses have, however, been given. For infants 3 grains may be administered thrice daily. As it produces no gastric or renal irritation, it is valuable as an intestinal antiseptic in diarrhœal diseases of children. It is prepared by Messrs. Knoll, of Ludwigshafen on Rhine.

